# The Radioprotective Activity of Resveratrol—Metabolomic Point of View

**DOI:** 10.3390/metabo12060478

**Published:** 2022-05-25

**Authors:** Michalina Gramatyka

**Affiliations:** Maria Skłodowska-Curie National Research Institute of Oncology, Gliwice Branch, Wybrzeże Armii Krajowej 15, 44-101 Gliwice, Poland; michalina.gramatyka@io.gliwice.pl

**Keywords:** metabolomics, radioprotection, resveratrol

## Abstract

Resveratrol, a plant-derived polyphenol, is an intensively studied compound with widely documented positive effects on health. Antioxidant activity is the property most often mentioned as responsible for its beneficial effects. Therefore, since the adverse effect of ionizing radiation is primarily related to the induction of oxidative stress, the question arises of whether the use of resveratrol could have a radioprotective effect. This paper summarizes the data on the cytoprotective activity of resveratrol and pieces of evidence for the potential interplay between response to radiation and resveratrol activity. The paper focuses on changes in the metabolic profile of cells and organisms induced by ionizing radiation and exposure to resveratrol. The comparison of metabolic changes induced by both factors provides a rationale for the potential mechanism of the radioprotective effects of resveratrol.

## 1. The Cytoprotective Activity of Resveratrol

Research on new substances that may have beneficial effects on human health is the objective of numerous studies. Natural compounds, commonly present in certain amounts in the standard diet, are a frequent subject of studies focused on their use in the prevention or treatment of various diseases. Among numerous plant-derived compounds, polyphenolic compounds are of great interest. (Poly)phenolic compounds have various functions in plants: they can be fragrances and colorants, poisons and feeding deterrents, allelopathic compounds, signaling molecules, structural components, and finally antifungal and antimicrobial agents [1]. Extensive literature data suggest that consumption of plant-derived polyphenols may have beneficial effects in reducing the development of many diseases, including cardiovascular, neurodegenerative, as well as cancer, which is most likely related to the antioxidant properties of this group of compounds [1,2,3]. There are over eight-thousand known polyphenolic compounds, divided into classes based on the structure of the carbon skeleton. The main classes include phenolic acids, flavonoids, stilbenes, tannins, and lignans [1,4].

Resveratrol naturally found in fruits, especially grapes, is probably the most-explored plant secondary metabolite ever. It belongs to the stilbene class and, like most stilbenes, is a phytoalexin synthesized by plants in response to damage or pathogens [4,5,6]. Numerous studies indicate that administration of resveratrol may result in reduced risk of cardiovascular disease (the so-called “French Paradox”) and obesity, exert anti-inflammatory, neuroprotective, and anti-aging effects, and may even reduce cancer risk and support cancer therapy [5,7,8,9].

Various cellular mechanisms putatively contribute to the beneficial effects of resveratrol. This includes antioxidant activity with an increase in the activity of enzymes responsible for counteracting oxidative stress (including glutathione peroxidase, heme oxygenase, and superoxide dismutase) [10,11,12]. Resveratrol also possesses the ability to alter the expression of numerous proteins, of which the most significant seems to be 5’ AMP-activated protein kinase (AMPK) and Sirtuin1 [13,14,15,16,17], i.e., proteins associated with lipids metabolism, ATP production, and overall regulation of cellular defense, including the cell cycle arrest in stress conditions [14,15,18,19]. Researchers also report the ability of resveratrol to increase the expression of NO synthase (eNOS), which results in vasodilation, reduction of hypoxia, and blood pressure [6,12,17]. Stone et al., suggest that the protective effect of resveratrol paradoxically results from its toxic effect on cells: exposure to low doses leads to the acquisition of stress-induced resilience resulting in the increased efficiency of self-repair mechanisms and overall improvement in health and longevity [20]. Such a statement is consistent with reports suggesting that resveratrol may act in hormesis. Lower doses (associated with oral intake) have beneficial effects, but at higher doses (which are achievable only in in vitro studies because of its pharmacokinetics) resveratrol displays toxic effects. Such mechanisms of action may be related to its action as a topoisomerase II poison [21,22].

## 2. Resveratrol Is a Radioprotective Compound

The primary mechanism of toxicity of ionizing radiation is its ability to induce oxidative stress in tissues, which directly and indirectly leads to damage to cell structures resulting in their improper functions [11,23,24]. The response of the biological system to ionizing radiation can be classified into one of three categories depending on the observed effect. (1) Radiosensitivity is directly attributed to cell damage and death, usually after exposure to high doses of radiation. (2) Radiosusceptibility describes the susceptibility of tissue to radiation-induced cancers, the appearance of which is associated with cellular transformation and genomic instability due to mis-repaired DNA damage. (3) Radiodegeneration is associated with accelerated senescence due to unrepaired and accumulating DNA damage, which, however, does not induce (instant) cell death or cancer transformation [25,26]. This third type of response seems to be involved in the phenomenon of radiation-induced late cardiotoxicity. Even relatively low doses of radiation, with no immediate effect on biological system dysfunction, can increase the risk of cardiac complications, usually ischemic heart disease, although the time between exposure and the onset of clinical symptoms can be very long [27,28,29]. The significance of this phenomenon is growing due to increased risk of exposure from medical as well as environmental and occupational sources and increased life expectancy, which in turn translates into an increased probability of detecting the late effects of radiation [27,28].

At low, “physiological” concentrations, reactive oxygen species (ROS) serve multiple functions: they act as signaling molecules, regulate physiological processes, participate in signal transduction, and activate ion channels and pathways involved in the acquisition of stress-induced resilience [30]. However, the excess of free radicals leads to oxidative stress, causes inflammatory reactions, lipid peroxidation, damage to cell structures (in particular cell membranes), and eventually cell death. The literature frequently highlights the damage to mitochondrial function and structure caused by ionizing radiation and ROS, which in the case of organs with high energy demands (such as the heart) can affect the proper functioning of the whole system [31].

Because one of resveratrol’s mechanisms of action is its antioxidant activity, researchers seek to answer the question of whether it may also have radioprotective properties. In a study on bone marrow cells isolated from irradiated mice, Carsten et al., showed that administration of resveratrol reduces the number of chromosomal lesions induced by radiation [19]. A similar result was obtained by Zhang et al., who observed reduced oxidative stress after total body irradiation (TBI) of mice and significant recovery of hematopoietic progenitor cells and hematopoietic stem cells in bone marrow after administration of resveratrol [32]. Furthermore, when mice were fed with resveratrol in doses equivalent to those taken orally as pills by humans (7 and 28 mg/kg), it led to a reduction in the number of micronuclei induced by radiation in reticulocytes derived from peripheral blood and bone marrow [11]. In a study on lymphocytes isolated from the blood of irradiated mice, resveratrol reduced the amount of DNA damage evaluated by comet assay [33]. Chromosome protection against radiation-induced damage was also observed in human lymphocytes isolated from irradiated blood samples after incubation with resveratrol at a dose of 2.2 µM [21]. However, the beneficial effect was no longer seen when a higher dose of resveratrol was administered (above 20 µM) [22], which may be related to the resveratrol influence on histone deacetylase activity by modification of Sirt1 expression. At a dose of 10 µM, resveratrol exerted a protective effect on irradiated mouse embryonic stem cells by improving their viability and enhancing DNA damage repair [34]. Simsek et al., showed that “physiological” doses of resveratrol (10 mg/kg) protect rat ovaries from radiation-induced damage, probably by stimulating the body’s natural antioxidant mechanisms [35]. The protective effect of resveratrol was also obtained in irradiated mouse testes [36]. Moreover, administration of resveratrol at 40 mg/kg/d protects the intestines from radiation damage, which was attributed to the superoxide dismutase 2 activation, most likely in a Sirt1-dependent manner [16]. Resveratrol also possesses neuroprotective activity, as demonstrated by Prager et al., who observed a positive effect of resveratrol on the number of nestin-positive neural progenitor cells following irradiation [37]. Table 1 summarizes the results of studies on the radioprotective activity of resveratrol.

Noteworthy, the potential cardioprotective activity of resveratrol is also addressed. Questions related to the putative radioprotective activity of resveratrol in cardiac tissue may be of great importance since even low doses of radiation, though considered harmless for decades, may have adverse health effects due to increased risk of cardiovascular malfunction [38]. However, surprisingly little is known about the cardioprotective potential of resveratrol when radiation is the harmful factor. Only a few studies analyzed the effect of resveratrol (or resveratrol-containing mixtures) on cardiovascular damage induced by ionizing radiation. This includes the study of DeFreitas et al., who described the effect of black grape juice on heart damage after total body irradiation on rats [24]. The authors have found that supplementation with black grape juice significantly reduced lipid peroxidation and mitochondrial damage in hearts after acute irradiation. However, because black grape juice is a mixture of several components with documented biological activity (including resveratrol, quercetin, rutin, caffeic and gallic acids, and catechin), it is impossible to conclude whether the protective effect was the result of a single compound (e.g., resveratrol) or a mixture.

## 3. Metabolomics and Radiation-Induced Changes in Cellular Metabolism

Metabolomics is a technique that allows the identification and quantitative measurement of metabolites in biological systems. It is one of the most effective methods to detect cellular response to various endogenous and exogenous (environmental) factors, including ionizing radiation [39]. Several metabolomics studies showed that even putatively harmless doses of radiation (up to 2 Gy) induce molecular changes that affect the metabolic profile. In general, radiation-induced changes involve disturbances in energy production (likely due to radiation-induced damage to mitochondria), lipid metabolism, and protein degradation. Moreover, levels of metabolites associated with oxidative stress and inflammation also change in irradiated tissues [31]. 

The analysis of radiation-altered metabolites allows the identification of specific metabolic pathways affected by radiation exposure. Table 2 summarizes available information about metabolic pathways whose changes were detected in different experimental models. In general, alterations in glutathione metabolism, amino acid metabolism, taurine/hypotaurine metabolism, and glyoxylate/dicarboxylate metabolism are the most frequent in irradiated cells and tissues. Moreover, disruption of the citrate cycle (TCA cycle) and lipid metabolism is usually observed in irradiated organisms. The detailed information on radiation-related changes in cellular metabolism could be found in other review papers (including [31]).

## 4. Resveratrol-Induced Changes in Cellular Metabolism

The vital importance of radiation-affected metabolic pathways it is important to address the question: what is the influence of resveratrol on cellular metabolism? However, the actual effect of resveratrol on the metabolism of cells and tissues remains a poorly studied field. The knowledge of mechanisms of radiation injury suggests that the potential radioprotective effect of resveratrol would likely involve the protection of mitochondrial function and energy metabolism. Hence, one might expect that the resveratrol administration would affect the metabolites associated with increased efficiency of energy production or improved antioxidative mechanisms since such changes will potentially antagonize the toxic effects of radiation. 

The few papers addressing this topic focus primarily on rodents. Mass spectrometry of plasma from rats subjected to trauma-hemorrhage (TH) revealed that resveratrol administration improves energy metabolism and reduces protein degradation. The specific changes included an increase in carnitine and a decrease in acetylcarnitine, butyrylcarnitine, trimethyl lysine, pipecolic acid, 3-ketobutyrate, 3-hydroxybutyrate, lactate, and citrate levels in TH with resveratrol supplementation group when compared to TH only group [54]. Another study in rats identified changes in the urinary metabolites using NMR spectroscopy. Within the first 12 h after resveratrol treatment, a decrease in hippurate, glycoproteins, taurine pantothenate, 2-oxoglutarate, alanine, creatinine, phenyl acetyl glycine, and trimethylamine N-oxide was observed, while levels of dimethylglycine and proline-betaine increased. Later on, 24 h after resveratrol administration, changes in metabolic profile were reduced and included a decrease in levels of hippurate and 2-oxoglutarate, as well as an increase in dimethylglycine, transaconitate, and taurine. Observed changes were linked to the resveratrol influence on the intestinal microbiome and associated with the reduction of oxidative stress and inflammation [55]. Another in vivo study addressed metabolite profiles in abdominal muscles of mice fed with a high-fat diet complemented with resveratrol. Mass spectrometry analysis revealed that resveratrol treatment influenced carbohydrate, amino acid, and lipid metabolisms (affected metabolites were involved in galactose, alanine, aspartate, glutamate, glyoxylate, and dicarboxylate metabolism). Noteworthy, reduced lipid accumulation was also observed in animals whose high-fat diet was complemented with resveratrol, which suggested the potential of resveratrol to protect against the development of atherosclerosis [56].

A particularly interesting human study explored the effects of resveratrol on metabolic profile in men with metabolic syndrome [57]. After 4 months of resveratrol supplementation, metabolic profiles of blood, urine, adipose tissue, and skeletal muscle tissue were measured using mass spectrometry. Changes in steroid hormones, sulfated androgen precursors, and long-chain saturated, monounsaturated, and polyunsaturated fatty acids were the most pronounced after resveratrol administration. The authors suggested that the observed changes may indicate the ability of resveratrol to influence the gut microbiome and to stimulate the conversion of fatty acids (mainly alpha-linolenic acid and linoleic acid) to polyunsaturated fatty acids (eicosapentaenoic acid and docosahexaenoic acid) by decreasing the amounts of androgen precursors due to their increased urinary excretion.

The influence of resveratrol on cellular metabolism was also analyzed using in vitro models. The study on fibroblasts with mitochondrial Complex I disorder revealed that resveratrol administration resulted in a reduction in polyunsaturated fatty acids, phosphocholine, lactate, myo-inositol, and taurine levels, while the content of glutamate/glutamine, glycine, alanine, and BCAA increases [58]. Another study addressed resveratrol-induced changes in human breast cancer cell lines. The authors observed an increase in serine, methionine, arachidonic acid, tryptophan, serotonin, and kynurenine concentrations, cell-line-dependent changes in aspartic acid, glutamine, glycine, ornithine, putrescine, and spermidine levels as well as and reduction of prostaglandin E2 level [59]. 

Table 3 summarizes available information about metabolic pathways whose changes were detected in different experimental models upon the resveratrol stimulation. The most significant changes induced by resveratrol treatment concerned taurine/hypotaurine metabolism, amino acid metabolism, glyoxylate/dicarboxylate metabolism, glutathione metabolism, lipid metabolism, and citrate cycle (TCA cycle).

## 5. The Combined Effects of Ionizing Radiation and Resveratrol on Cellular Metabolism

There is only one report yet that addressed the influence of resveratrol administration on the metabolic profile of murine heart irradiated in vivo. The authors documented by NMR profiling that resveratrol supplementation changed levels of several metabolites in murine hearts 20 weeks after irradiation with a single 2 Gy dose. The administration of resveratrol mitigated the radiation-induced decline in the content of choline-containing compounds and unsaturated lipids, which might reflect the stabilization of cell membrane structure against radiation-related damage. Moreover, resveratrol itself affected metabolites associated with maintaining the balance of energy production—increased glycine and hypotaurine and decreased lactate levels. Obtained results fit the concept that resveratrol supplementation may prevent metabolic changes related to radiation-induced damage to the heart [44].

**Figure 1:**
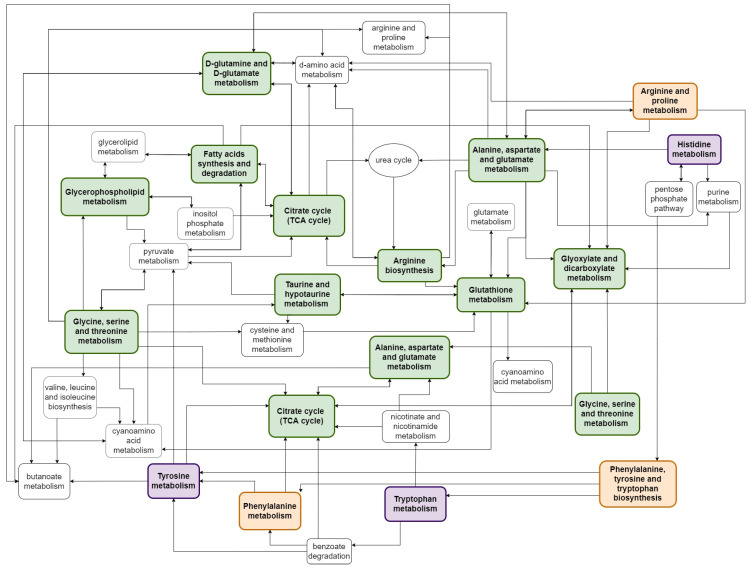
Cellular metabolic pathways affected by ionizing radiation and resveratrol. The diagram illustrates the connections between selected pathways modulated by resveratrol (purple boxes), radiation (orange boxes), and both factors (green boxes).

**Figure 2:**
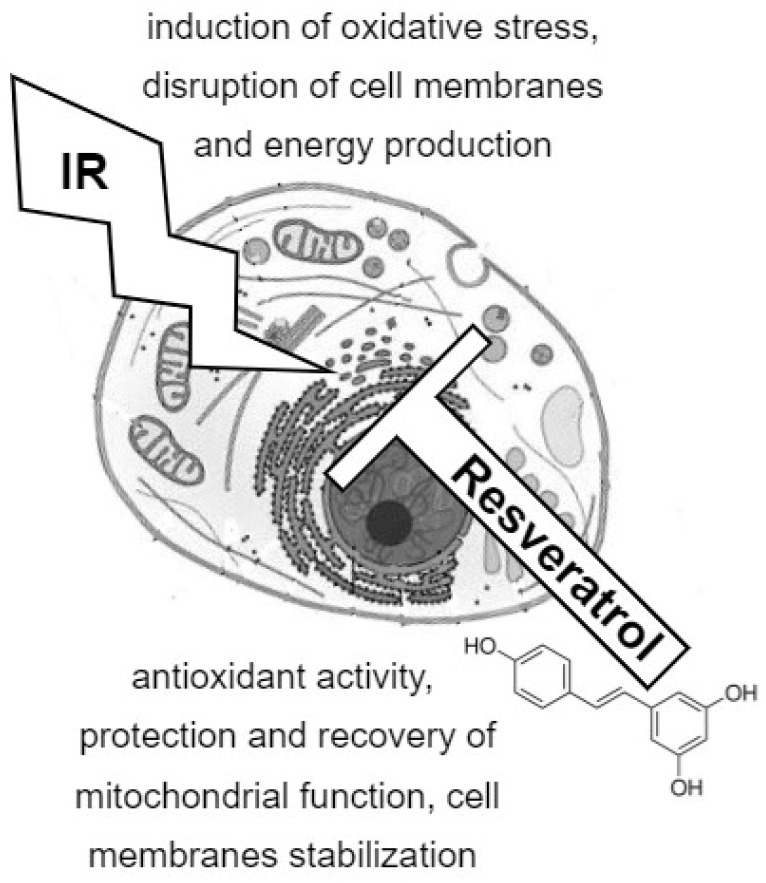
Hypothetical metabolism-related mechanism of radioprotection by resveratrol: main mechanisms of radiation toxicity and protective effects of resveratrol. IR—ionizing radiation.

Nevertheless, when the comparative analysis of metabolic pathways altered by exposure to radiation and resveratrol was performed, several common pathways could be identified that are illustrated in Figure 1. Mechanisms commonly affected by radiation and resveratrol involve the metabolism of lipids, including the metabolism of fatty acids and phospholipids. However, it has been suggested that irradiation leads to lipid degradation, whereas resveratrol is believed to have beneficial effects on fatty acid metabolism and the stability of lipid membranes. Both ionizing radiation and resveratrol affect glutathione metabolism, which translates into the antioxidant potential of cells. Moreover, both factors changed levels of compounds participating in the citrate cycle, which affected energy production in the cell. These basic mechanisms related to radiation toxicity and radioprotective action of resveratrol are schematically presented in Figure 2. The other pathways affected by both ionizing radiation and resveratrol include the metabolism of different amino acids (alanine, aspartate, arginine, glutamine, glutamate, glycine, serine, threonine, etc.,) as well as the metabolism of taurine/hypotaurine, glyoxylate, and dicarboxylate. It seems probable that the “common part” of cellular metabolism is involved in the radioprotective properties of resveratrol. However, more experimental data are required to confirm that radiation and resveratrol affected the same metabolic pathways in defined biological models having the opposite influence on key metabolites involved in cell homeostasis, energy production, and antioxidant potential.

## 6. Conclusions

Resveratrol, a well-recognized dietary compound, possesses numerous beneficial health properties. An important area of its possible application is radioprotection, that is, counteracting and protecting against the negative effects of radiation exposure. To date, resveratrol has been shown to have radioprotective effects on bone marrow and germ cells, as well as on neuronal cells. There are also single reports of radioprotective effects of resveratrol in the context of the heart muscle. However, molecular mechanisms of radioprotective activity of resveratrol remain to be identified. The use of metabolic profiling techniques—NMR spectroscopy and mass spectrometry—enables the detection of molecular changes induced either by radiation or natural products. Therefore, a metabolomics approach could be implemented to explore the detailed mechanisms of resveratrol bioactivity. The results of available metabolomic studies indicate that the beneficial effects of resveratrol could be associated, at least in part, with the “counteracting” metabolic effects of radiation (which is schematically presented in Figure 2). For example, ionizing radiation disrupts proper mitochondrial function, whereas resveratrol may have beneficial effects on the function and structure of mitochondria. However, specific mechanisms responsible for the protective action of resveratrol require further metabolomic studies that would address several important yet unanswered questions. For example, it is unclear whether a hypothetical effect of resveratrol is associated solely with prior preconditioning or administration of this compound after irradiation would also produce any effect, which is crucial from the perspective of resveratrol used in radioprotection.

## Figures and Tables

**Table 1:** An overview of studies on the radioprotective effects of resveratrol.

Research Model	Resveratrol Dose	Radiation Dose	The Observed Effect of Resveratrol	Reference
bone marrow cells from CBA/CaJ irradiated mice	100 mg/kg/day from 2 days before the irradiation until the end of the experiment	3 Gy, γ radiation	2.8-fold reduction of total chromosome aberrations, including gaps, dicentrics, and Robertsonian translocations for the resveratrol + radiation group compared to the radiation group	[19]
peripheral blood cells and bone marrow cells from irradiated C57BL/6-Ly-5.1 mice	20 mg/kg/day from 7 days before to 30 days after irradiation	6.0 Gy or 7.2 Gy, 137 Cs irradiator, TBI	increased survival after TBI, decreased acute and long-term bone marrow damage, reduced oxidative stress after exposure to 7.2 Gy in the resveratrol group	[32]
blood and bone marrow from irradiated Swiss mice	7 mg/kg/day or 28 mg/kg/day for 2 weeks	5 Gy and 10 Gy total doses in 0.5 Gy and 1 Gy fractions, X-radiation, TBI	reduction in the number of micronuclei in reticulocytes in the resveratrol + radiation group when compared to the radiation group	[11]
peripheral blood lymphocyte from irradiated NMRI mice	50 mg/kg or 100 mg/kg 2 h before irradiation	2 Gy, γ radiation	reduction of radiation-induced DNA damage (assessed by comet assay) in the resveratrol group	[33]
human peripheralblood lymphocytes	2.2, 22 or 220 µM 1 h before irradiation	2 Gy γ radiation	reduction in chromosome aberrations after irradiation with maximal protection observed for 2.2 µM dose; however, resveratrol induced chromosomal aberrations in the absence of irradiation	[21]
human peripheral blood lymphocytes	20 µM or 40 µM 3 h before irradiation	0.5 Gy or 1 Gy, X-radiation	in the 40 µM resveratrol group increased level of dicentric chromosomes induced by radiation; resveratrol alone did not induce DNA or chromosome damage	[22]
mouse embryonic stem cells	10 µM 48 h before irradiation	5 Gy, X-radiation	improvement of the viability of irradiated cells and acceleration of DNA damage repair	[34]
ovaries from irradiated Wistar rats	10 mg/kg or 100 mg/kg 24 h before irradiation	720 cGy, photon, TBI	increased follicle count in ovaries after irradiation and increase of antioxidant enzymes activity in the resveratrol group	[35]
testes from irradiated NMRI mice	100 mg/kg/day for two days before irradiation	2 Gy, γ radiation, TBI	reduction of spermatogenic arrest, thickening of the basal lamina, decreased sperm density and vacuolation in the resveratrol group; resveratrol increased atrophy of seminiferous tubules	[36]
small intestines of irradiated C57BL/6 N mice	40 mg/kg/day1 day before and 5 days after irradiation	7 Gy, 137 Cs irradiator, partial-body irradiation	normalization of the intestinal cell morphology in irradiated mice (enhanced regeneration of intestinal crypt cells, increased villi length, shorter basal lamina length)	[16]
organotypic entorhinal–hippocampal slice cultures generated from nestin-CFPnuc C57BL/J6 mice	15 µM 2 h before irradiation until 48 h after irradiation	4.5, 8, 12, or 16 Gy, X-radiation	increased number of nestin-positive neural progenitor cells in the resveratrol + radiation group when compared to the radiation group	[37]

**Table 2:** Radiation-induced modulation of metabolic pathways. Pathways were identified using the MetaboAnalyst 5.0 software based on radiation-affected metabolites.

Experimental Model	Radiation Dose and Experimental Design	Affected Metabolic Pathway	Reference
murine liver	3 Gy and 7.8 Gy, proton and gamma, 4 and 11 days	GSH metabolism; Ala/Asp/Glu metabolism; Gly/Ser/Thr metabolism; TCA cycle; Glycerophospholipid metabolism; Pyruvate metabolism	[40]
rat jejunum, spleen, liver and plasma	2 Gy and 6 Gy X-ray, 1, 2, and 3 days	Gln/Glu metabolism; Phe/Tyr/Trp biosynthesis; Taurine and hypotaurine metabolism; Ala/Asp/Glu metabolism; GSH metabolism; Phe metabolism; Gly/Ser/Thr metabolism; Glyoxylate and dicarboxylate metabolism; Arg biosynthesis; TCA cycle; Arg/Pro metabolism; Glycerophospholipid metabolism; Primary bile acid biosynthesis	[41]
cardiomyocytes	2 Gy, photons, 2 days	Taurine and hypotaurine metabolism; Gln/Glu metabolism; GSH metabolism; Gly/Ser/Thr metabolism; Ala/Asp/Glu metabolism; Glyoxylate and dicarboxylate metabolism; Arg biosynthesis; Glycerophospholipid metabolism	[42]
murine hearts	2 Gy, photons, 2 days, 20 weeks	Gln/Glu metabolism; Phe/Tyr/Trp biosynthesis; Ala/Asp/Glu metabolism; Gly/Ser/Thr metabolism; Glyoxylate and dicarboxylate metabolism; Arg biosynthesis; GSH metabolism; Inositol phosphate metabolism; Tyr metabolism	[43]
murine hearts	2 Gy, photons, 20 weeks	Glycerophospholipid metabolism; Lipids metabolism	[44]
whole mice, 31P NMR MRI	7 Gy, X-ray, 0–14 days	Arg/Pro metabolism; Gly/Ser/Thr metabolism	[45]
murine urine	8 Gy, X-ray, 7 days	Taurine and hypotaurine metabolism; TCA cycle; Ala/Asp/Glu metabolism; Butanoate metabolism; Gly/Ser/Thr metabolism; Gln/Glu metabolism; Phe metabolism; Arg biosynthesis; Propanoate metabolism; Glyoxylate and dicarboxylate metabolism; Glycerophospholipid metabolism; Arg/Pro metabolism; Primary bile acid biosynthesis	[46]
fibroblasts	1 Gy and 5 Gy, gamma, 1, 2, and 3 days	Lipids metabolism; Phe/Tyr/Trp biosynthesis; Phe metabolism; GSH metabolism; Arg/Pro metabolism; Glycerophospholipid metabolism; Arg biosynthesis; Aminoacyl-tRNA biosynthesis; Ubiquinone and another terpenoid-quinone biosynthesis; Pantothenate and CoA biosynthesis; Ether lipid metabolism; Gly/Ser/Thr metabolism; Cys/Met metabolism; Trp metabolism; Tyr metabolism	[47]
fibroblasts, B lymphoblastoid cells	0.02 Gy, 0.1 Gy, and 1 Gy, X-ray, 1 and 10 h.	Purine metabolism; Cys/Met metabolism	[48]
murine urine	1.1 Gy and 4.4 Gy, X-ray, 2 days	intermediates in the Trp metabolism and Ile catabolism; TCA cycle; Pyruvate metabolism; Glycolysis/Gluconeogenesis; Ala/Asp/Glu metabolism; Glyoxylate and dicarboxylate metabolism; Cys/Met metabolism	[49]
murine liver	8.5 Gy, gamma, 1 and 4 days	Lipids metabolism; GSH metabolism; Porphyrin and chlorophyll metabolism; Pyrimidine metabolism; Glycerophospholipid metabolism; Primary bile acid biosynthesis; Purine metabolism	[50]
murine intestines	2 Gy or 1.6 Gy, gamma or heavy-ion, 2 months	Phe/Tyr/Trp biosynthesis; Phe metabolism; Ala/Asp/Glu metabolism; beta-Ala metabolism; His metabolism; Pyruvate metabolism; GSH metabolism; Trp metabolism; Pyrimidine metabolism; Glycolysis/Gluconeogenesis; Pantothenate and CoA biosynthesis; TCA cycle	[51]
bone marrow, ileum, liver, muscle, lung, serum, urine of mice	6 Gy, gamma, 12 h	Gln/Glu metabolism; Taurine and hypotaurine metabolism; Phe/Tyr/Trp biosynthesis; GSH metabolism; Ala/Asp/Glu metabolism; Phe metabolism; Purine metabolism; Arg biosynthesis; Pyrimidine metabolism; Arg/Pro metabolism; Glycerophospholipid metabolism; Amino sugar and nucleotide sugar metabolism; Primary bile acid biosynthesis; Pentose and glucuronate interconversions	[52]
urine and serum of rhesus monkeys	4 Gy, gamma, up to 60 days	Phe/Tyr/Trp biosynthesis; TCA cycle; Phe metabolism; Glyoxylate and dicarboxylate metabolism	[53]

GSH—glutathione; CoA—coenzyme A; amino acids; Ala—alanine; Arg—arginine; Asn—asparagine; Asp—aspartate; Cys—cysteine; Gln—glutamine; Glu—Glutamate; Gly—glycine; Ile—isoleucine; Met—methionine; Phe—phenylalanine; Pro—proline; Ser—serine; Tyr—tyrosine, Trp—tryptophan.

**Table 3:** Resveratrol-related changes in metabolic pathways. Pathways were identified using the MetaboAnalyst 5.0 software based on resveratrol-affected metabolites.

Experimental Model	Resveratrol Dose and Experimental Design	Affected Metabolic Pathway	Reference
plasma from Sprague –Dawley rats subjected to trauma-hemorrhagic shock	30 mg/kg administered 30 min after hemorrhage	Synthesis and degradation of ketone bodies; His metabolism; Butanoate metabolism; TCA cycle; Glyoxylate and dicarboxylate metabolism; Lys degradation; Aminoacyl-tRNA biosynthesis; Biotin metabolism; beta-Ala metabolism; Pyruvate metabolism; Ala/Asp/Glu metabolism; Val/Leu/Ile degradation; Glycolysis/Gluconeogenesis; Tyr metabolism	[54]
urine and feces of Wistar rats	50 mg/kg or 250 mg/kg after 12 h of food deprivation	Taurine and hypotaurine metabolism; Pyruvate metabolism; TCA cycle; Gly/Ser/Thr metabolism; Ala/Asp/Glu metabolism; Glycolysis/Gluconeogenesis	[55]
abdominal muscle tissue from ApoE-/- mice fed with a high fat diet	10 mg/kg/day for 24 weeks	Pentose phosphate pathway; pentose and glucuronate interconversions; galactose metabolism; fructose and mannose metabolism; Ala/Asp/Glu metabolism; Glyoxylate and dicarboxylate metabolism	[56]
blood, urine, adipose tissue, and skeletal muscle from men with metabolic syndrome	150 mg/day or 1 g/day for 4 months	Linoleic acid metabolism; Ubiquinone and another terpenoid-quinone biosynthesis; His metabolism, Tyr metabolism; Trp metabolism; Biosynthesis of unsaturated fatty acids; Phe/Tyr/Trp biosynthesis	[57]
fibroblasts with mitochondrial Complex 1 disorder	50 µM for 24 h	Lipids transformations; Gln/Glu metabolism; Taurine and hypotaurine metabolism; Ala/Asp/Glu metabolism; Gly/Ser/Thr metabolism; Glyoxylate and dicarboxylate metabolism; Arg biosynthesis; GSH metabolism; Inositol phosphate metabolism; Arg/Pro metabolism; Aminoacyl-tRNA biosynthesis; Val/Leu/Ile biosynthesis; Val/Leu/Ile degradation; Nitrogen metabolism	[58]
MCF-7 and MDA-MB-231 breast cancer cells	100 µM for 72 h	Phe metabolism; Arg biosynthesis; Ala/Asp/Glu metabolism; Gln/Glu metabolism; Arg/Pro metabolism; Taurine and hypotaurine metabolism; Phe metabolism; Trp metabolism; Arachidonic acid metabolism; His metabolism; Gly/Ser/Thr metabolism; GSH metabolism; Glyoxylate and dicarboxylate metabolism; Cys/Met metabolism; Tyr metabolism; Aminoacyl-tRNA biosynthesis; Val/Leu/Ile biosynthesis; beta-Ala metabolism; Lipids transformations	[59]
hearts from irradiated C57Bl/6NCrl mice	5 mg/kg/day or 25 mg/kg/day from 4 weeks before until 2 weeks after irradiation	Lipids transformations; Gly/Ser/Thr metabolism; Taurine and hypotaurine metabolism; Glyoxylate and dicarboxylate metabolism; Glycerophospholipid metabolism; GSH metabolism; Primary bile acid biosynthesis	[44]

GSH—glutathione; CoA—coenzyme A; amino acids; Ala—alanine; Arg—arginine; Asn—asparagine; Asp—aspartate; Cys—cysteine; Gln—glutamine; Glu—Glutamate; Gly—glycine; His – histidine; Ile—isoleucine; Leu—leucine; Met—methionine; Phe—phenylalanine; Pro—proline; Ser—serine, Tyr—tyrosine; Trp—tryptophan; Val—valine.
